# Validation of the Korean version of the pediatric quality of life inventory™ 4.0 (PedsQL™) generic core scales in school children and adolescents using the rasch model

**DOI:** 10.1186/1477-7525-6-41

**Published:** 2008-06-02

**Authors:** Seung Hee Kook, James W Varni

**Affiliations:** 1 Department of Psychiatry, Chonnam National University Hospital, 8 Hak-dong, Dong-gu, Gwangju 501-757, South Korea; 2 Department of Pediatrics, College of Medicine, Department of Landscape Architecture and Urban Planning, College of Architecture, Texas A&M University, 3137 TAMU, College Station, TX 77843-3137, USA

## Abstract

**Background:**

The Pediatric Quality of Life Inventory™ (PedsQL™) is a child self-report and parent proxy-report instrument designed to assess health-related quality of life (HRQOL) in healthy and ill children and adolescents. It has been translated into over 70 international languages and proposed as a valid and reliable pediatric HRQOL measure. This study aimed to assess the psychometric properties of the Korean translation of the PedsQL™ 4.0 Generic Core Scales.

**Methods:**

Following the guidelines for linguistic validation, the original US English scales were translated into Korean and cognitive interviews were administered. The field testing responses of 1425 school children and adolescents and 1431 parents to the Korean version of PedsQL™ 4.0 Generic Core Scales were analyzed utilizing confirmatory factor analysis and the Rasch model.

**Results:**

Consistent with studies using the US English instrument and other translation studies, score distributions were skewed toward higher HRQOL in a predominantly healthy population. Confirmatory factor analysis supported a four-factor and a second order-factor model. The analysis using the Rasch model showed that person reliabilities are low, item reliabilities are high, and the majority of items fit the model's expectation. The Rasch rating scale diagnostics showed that PedsQL™ 4.0 Generic Core Scales in general have the optimal number of response categories, but category 4 (almost always a problem) is somewhat problematic for the healthy school sample. The agreements between child self-report and parent proxy-report were moderate.

**Conclusion:**

The results demonstrate the feasibility, validity, item reliability, item fit, and agreement between child self-report and parent proxy-report of the Korean version of PedsQL™ 4.0 Generic Core Scales for school population health research in Korea. However, the utilization of the Korean version of the PedsQL™ 4.0 Generic Core Scales for healthy school populations needs to consider low person reliability, ceiling effects and cultural differences, and further validation studies on Korean clinical samples are required.

## Background

Health-related quality of life (HRQOL) measures should be based on patient's perceptions through self-assessment, use understandable and age appropriate language, provide evidence of acceptable or good reliability and validity, assess multiple dimensions, and consist of a 'core' set of questions as well as a set of specific items for different conditions. In addition, HRQOL measures should be feasible; that is, they should be short so that they may be administered repeatedly and easy to score and analyze, be acceptable to patients by being inoffensive, and be usable in a busy, clinical setting. Patients who are ill become tired after 15–20 minutes and lengthy questionnaires can increase the risk of failure to complete them or items near the end of a questionnaire [1].

The assessment of pediatric HRQOL is complicated by developmental considerations and by questions regarding the accuracy and acceptability of parent-proxy ratings of patients' quality of life. The Pediatric Quality of Life Inventory™ (PedsQL™) is a measure with demonstrated reliability and validity for child self-report and parent proxy-report. It has been developed to assess HRQOL in children and adolescents from 2 to 18 years of age. It is based on a modular approach with generic and disease-specific instruments. As a generic instrument, the PedsQL™ 4.0 Generic Core Scales are brief (23 items), practical (less than 4 minutes to complete), flexible (designed for use with community, school, and clinical pediatric populations), and multidimensional [2]. The PedsQL™ 4.0 Generic Core Scales cover physical, emotional and social functioning which are the core dimensions of health as delineated by the World Health Organization (WHO), as well as role (school) functioning.

The PedsQL™ 4.0 Generic Core Scales have previously demonstrated evidence of feasibility, reliability and validity as a school population health measure in a U.S. sample [3], as well as in numerous clinical populations [4-10]. These previous studies have demonstrated the reliability and validity of PedsQL™ 4.0 Generic Core Scales using Classical Test Theory (CTT). However, CTT has a limitation that it is unable to estimate item difficulty and person ability characteristics separately. Another limitation of CTT is that it yields only a single reliability estimate and corresponding standard error of measurement, but the precision of measurement varies by ability level. Because of these limitations, the CTT method is less than ideal for applications that require item difficulty, person ability, and conditional standard error of measurement [11].

Although CTT has served test development well over several decades, Item Response Theory (IRT) has rapidly become mainstream as the theoretical basis for measurement [12]. IRT methods model the association between a respondent's underlying level on a characteristic (latent variable) and probability of a particular item response using a non-linear monotonic function [13]. The Rasch model [14], sometimes referred to as a one-parameter logistic model under IRT, provides a mathematical framework against which test developers can compare their data. The model is based on the idea that useful measurement involves examination of only one human attribute at a time (unidimensionality) on a hierarchical "more than/less than" line of inquiry. Person and item performance deviations from that line (fit) can be assessed, alerting the investigator to reconsider item wording and score interpretations from these data [15]. Additionally, the way each rating scale is constructed has great influence on the quality of data obtained from the scale [16], and a rating scale may not be used by respondents in the way it was intended by the developer of the scale [15]. Thus, the assumptions about both the quality of the measures and utility of the rating scale in facilitating interpretable measures should be tested empirically [15], which can be done utilizing the Rasch model [17].

The PedsQL™ 4.0 Generic Core Scales have been linguistically validated in many different languages. However, only local translations without linguistic validation have been available in Korea [18]. This study aimed to assess the psychometric properties of the Korean translation of the PedsQL™ Generic Core Scales for Korean school children and adolescents. The feasibility, reliability, construct validity, and agreement between child self-report and parent proxy-report were investigated based on previous PedsQL™ 4.0 CTT methods [3,6-10]. Additionally, the person and item reliability, item statistics and category functioning were assessed using the Rasch model [17].

## Methods

### Participants and settings

The Korean translations of PedsQL™ 4.0 Generic Core Scales were administered to schoolchildren ages 8–18 and their parents in 60 classes (28 elementary school classes, 16 middle school classes, and 16 high school classes) at 5 elementary schools, 5 middle schools, and 4 high schools within two small cities, two metropolitan cities, and a capital city. Classes at schools were randomly selected within grade. Trained research personnel visited each classroom and distributed the questionnaires and informed parent consent and child assent forms for students to take home to their parents. Parents signed the informed consent and completed the parent report surveys at home, then returned them to school via students. Parents were asked to return the surveys even if they chose not to consent to participate. The students completed their questionnaire after the parents gave informed consent. The consent rate of all classes was above 70%.

### Measures

#### The Korean translations of the Pediatric Quality of Life Inventory™ Version 4.0(PedsQL™ 4.0) Generic Core Scales

The 23-item PedsQL™ 4.0 Generic Core Scales encompass: (1) Physical functioning (8 items), (2) Emotional functioning (5 items), (3) Social functioning (5 items), and (4) School functioning (5 items). The PedsQL™ 4.0 Generic Core Scales are composed of parallel child self-report and parent proxy-report formats. Child self-report includes ages 5–7, 8–12, and 13–18. Parent proxy-report includes ages 2–4 (toddler), 5–7 (young child), 8–12 (child), 13–18 (adolescent), and assesses parent's perception of their child's HRQOL. The items for each of the forms are essentially identical, differing in the developmentally appropriate language, or first or third person tense. The instructions ask how much of a problem each item has been during the past 1 month. A 5-point response scale is utilized across child self-report for ages 8–18 and parent proxy-report (0 = never a problem; 1 = almost never a problem; 2 = sometimes a problem; 3 = often a problem; 4 = almost always a problem). Items are reverse-scored and linearly transformed to 0–100 scale (0 = 100, 1 = 75, 2 = 50, 3 = 25, 4 = 0), so that higher scores indicate better HRQOL. Scale scores are computed as the sum of the items divided by the number of items answered (this accounts for missing data). The physical health summary score is the same as the physical functioning subscale. To create the psychosocial health summary score, the mean is computed as the sum of the items divided by the number of items answered in the emotional, social, and school functioning subscales. If more than 50% of the items in a scale are missing, the Scale Score is not computed [3,19].

The PedsQL™ 4.0 Generic Core Scales were translated independently into Korean by a clinical psychologist and a social psychologist fluent in English and translated back into English by a bilingual English native speaker. After review and comments by the instrument author, the second Korean translations of the PedsQL™ 4.0 Generic Core Scales were tested on a panel of 13 school children with cognitive interviewing methods. The cognitive interviews were conducted by four certified clinical psychologists at the participant's home and revisions in the translation were conducted to rectify the identified problems. Finally, the third versions were produced and proofread to be considered as final. All the results of phases were reported to the instrument author and Mapi Research Institute, which were reviewed and accepted by them.

#### The Korean translation of the PedsQL™ Family Information Form

The PedsQL™ Family Information Form [10] was completed by parents. The PedsQL™ Family Information Form contains demographic information including the child's date of birth, gender, race/ethnicity, and parental education and occupation information. One survey question asks the parent to report on the presence of a chronic health condition ("In the past 6 months, has your child had a chronic health condition?") defined as a physical or mental health condition that has lasted or is expected to last at least 6 months and interferes with the child's activities. If the parents check "Yes" to this question, they are asked to write in the name of the chronic health condition.

This form also was translated independently into Korean by two clinical psychologists fluent in English and translated back into English by a bilingual English native speaker. After review and comment by the instrument author, the Korean translations of the PedsQL™ Family Information Form was revised and accepted by the instrument author. All the results of phases were reported to the instrument author and Mapi Research Institute.

### Statistical analysis

The feasibility of the PedsQL™ 4.0 Generic Core Scales as a school health measure was determined from the percentage of missing values for each item and distribution of item responses [20,21]. Range of measurement was further tested based on the percentage of scores at the extremes of the scaling range, that is, the maximum possible score (ceiling effect) and the minimum possible score (floor effect) [21]. Scale descriptives for child self-report and parent proxy-report were calculated using SPSS Version 13.0 for Windows.

Factor structure of the PedsQL™ 4.0 Generic Core Scales across age group was examined by a confirmatory factor analysis (CFA) of items with missing data, using the software Mplus [22]. The missing data option in Mplus was implemented to avoid list-wise deletion. Factor indicators were stated as categorical variables due to ceiling effect and the estimator was weighted least square parameter estimates using a diagonal weighted matrix with standard errors and mean-and variance-adjusted chi-square test statistic (WLSMV). WLSMV is one of the estimators that are robust to non-normality and involves the analysis of a matrix of polychoric correlations. The PedsQL™ four-factor model was tested, which consisted of physical, emotional, social, and school functioning factor. Additionally, the PedsQL™ second-order factor model was tested, which consisted of physical health and psychosocial health factors. Psychosocial health factor was the second-order factor, which consisted of three first-order factors including emotional, social and school functioning factor. The physical health factor is the same as the Physical Functional Scale.

The fit of models was evaluated by Chi-square statistic and fit indices including the Comparative Fit Index (CFI) [23], Tuker-Lewis Index (TLI) [24], and Root Mean Square Error of Approximation (RMSEA) [25]. Chi-square is a test of exact fit. With large samples, there is considerable power to reject the null hypotheses, even though the model may fit the data well. Therefore, other goodness of fit indices should be considered. The CFI [23] and TLI [24] both are incremental fit indices, ranging from 0 (indicating poor fit) to 1.00 (indicating a perfect fit) and are derived from the comparison of a restricted model with a null model. For two indices, a value greater than .90 indicates a psychometrically acceptable fit to the data. More recent literature suggests that high values greater than or equal to .95 indicate a good fit [26]. RMSEA is one of absolute fit indices and a measure of discrepancy between the observed and model implied covariance matrices adjusted for degrees of freedom. The values of RMSEA of .05 or less indicate close fit, less than .08 indicates a fair or reasonable fit, less than .10 indicates a mediocre fit, and greater than .10 indicates an unacceptable fit [25].

Construct validity was further determined utilizing the known-groups method. The known-groups method compares scale scores across groups known to differ in the health construct being investigated. In this study, groups differing in health status (healthy vs. chronic health condition groups) were compared, using t-tests. In order to determine the magnitude of the differences between healthy children and children with chronic health conditions, effect sizes were calculated [27]. Effect size as utilized in these analyses was calculated by taking the difference between the healthy sample mean and the chronic health condition sample mean, divided by the healthy sample standard deviation.

The person and item reliability, item statistics, and category functioning were assessed by the Rasch rating scale model (RSM) [28], using WINSTEPS [29]. The Rasch RSM analyses were conducted on the four subscales of child self-report and parent proxy-report. The Rasch model [17] can be generalized to polytomous items with ordered categories. The formulation of an extended Rasch model includes Partial Credit Model (PCM) [30] and Rating Scale Model (RSM) [31]. Given that Likert scales can be modeled according to either a PCM or a RSM, it is necessary to determine which polytomous Rasch model and its respective set of estimated parameters would best explain the data. To choose an appropriate model, several estimates obtained from the PCM and RSM are compared on the scales. For this study, a more parsimonious model, the RSM was chosen because the two models produced comparable person and item fit, reliability estimates.

The person reliability indicates the replicability of person ordering we would expect if this sample of persons were to be given another set of items measuring the same construct [28]. Analogous to Cronbach's alpha, it is bounded by 0 and 1. Person separation index is an estimate of the spread or separation of persons on this measured variable. Item reliability index is the estimate of the replicability of item placement within a hierarchy of items along the measured variable if these same items were to be given to another sample of comparable ability. Analogous to Cronbach's alpha, it is bounded by 0 and 1. The item separation index is an estimate of the spread or separation of items on the measured variable. It is expressed in standard error units. The person and item separation should be at least 2, indicating that the measure separated persons, items, or both into at least two distinct groups [15].

To check if items fit the model's expectation, item fit mean square (MNSQ) statistics were computed using the RSM. MNSQ determines how well each item contributes to defining one common construct. Item MNSQ values of about 1.0 are ideal and values greater than 1.4 may indicate a lack of construct homogeneity with other items in a scale and item MMSQ values smaller than 0.6 may indicate item redundancy [32]. However, the cutoff values tend to vary depending on the purpose for which the ratings are used [33]. Typically, two MNSQ statistics are used: infit (weighted) and outfit (unweighted) statistics. Infit is more sensitive to misfitting responses to items near the person's ability level, while outfit is sensitive to misfitting items that are further away [34].

It is often the case that respondents fail to react to a rating scale in the manner the test constructor intended [35]. Because it is always uncertain how a rating scale was used by a sample, an investigation of the functioning of the rating scale is always necessary [36] and can be done with the Rasch analysis. The rating scale diagnostics include category frequencies, average measures, threshold estimates, probabilities, and category fit. These diagnostics should be used in combination [15]. Average measure are defined as the average of the ability estimates for all persons in the sample who choose that particular response category, with the average calculated across all observations in that category [37]. They increase monotonically, indicating that on average, those with higher abilities/stronger attitudes endorse the higher categories, whereas those with lower abilities/weaker attitudes endorse the lower categories [15]. Because observations in higher categories must be produced by higher measures, the average measures across categories must increase monotonically. Fit statistics provide another criterion for assessing the quality of rating scales. Outfit mean squares greater than 1.3 indicate more misinformation than information, meaning that the particular category is introducing noise into the measurement process. The step measures or thresholds define the boundaries between categories. Thresholds too should increase monotonically [38]. Thresholds not increasing monotonically across the rating scale are considered disordered [15].

Finally, agreement between child self-report and parent proxy-report was determined through two-way mixed effect model (absolute agreement, single measure) Intraclass Correlations (ICC) [39]. The ICC offers an index of absolute agreement given that it takes into account the ratio between subject variability and total variability [39,40]. Intraclass Correlations (ICC) are designated as ≤ 0.40 poor to fair agreement, 0.41–0.60 moderate agreement, 0.61–0.80 good agreement, and 0.81–1.00 excellent agreement [41]. Statistical analyses were conducted using SPSS Version 13.0 for Windows.

## Results

### Sample characteristics

The overall response rate was 70.9%. The response rate for the elementary school survey (grades three through six) was 71.0%. The response rate for the middle and high schools was 70.8. A total 1453 of parent-child dyads completed the Korean translations of PedsQL™ 4.0 Generic Core Scales and the Korean translations of PedsQL™ Family Information Form. Child self-reports for 1425 (98.1%) children were available since 28 (1.9%) child self-reports had more than 50% missing items in the scale. Parent proxy-reports for 1431 (98.5%) parents were available since 22 (1.5%) parent proxy-reports had more 50% missing items in the scale. There were 633 (44.4%) child self-reports and 638 (44.6%) parent proxy-reports for ages 8–12. There were 792 (55.6%) adolescent self-reports and 793 (55.4%) parent proxy-reports for ages 13–18.

The number of boys (n = 644, 45.2%) was less than the number of girls (n = 781, 54.8%; missing = 28, 1.9%). The race/ethnicity of the total sample was Asian. Respondents of parent self-report consisted of mother (n = 1250, 86.0%), father (n = 159, 10.9%), grandmothers (n = 5, 0.3%), grandfathers (n = 3, 0.2%), guardians (n = 1, 0.1%), and others (n = 12, 0.8%; missing = 23, 1.6%). Of the respondents, mothers' education level was 6^th ^grade or less (n = 16, 1.3%), 7^th ^through 9^th ^grade or less (n = 55, 4.4%), 10^th ^to 12^th ^grade or less (n = 609, 48.7%), some college or certification course (n = 153, 12.2%), college graduate (n = 358, 28.6%), graduate or professional degree (n = 32, 2.6%; missing = 27, 2.2%). Of the respondents, fathers' education level was 6^th ^grade or less (n = 4, 2.5%), 7^th ^through 9^th ^grade or less (n = 8, 5.5%), 10^th ^to 12^th ^grade or less (n = 55, 34.0%), some college or certification course (n = 13, 8.2%), college graduate (n = 64, 40.3%), graduate or professional degree (n = 11, 6.9%; missing = 5, 3.1%). The sample included 1396 (96.1%) healthy children and 50 (3.4%; missing = 7, 0.5%) children whose parents reported the presence of chronic health condition in the past 6 months.

### Feasibility

The percentage of missing item responses was less than 1.7% for child self-report and 1.4% for parent proxy-report.

### Descriptive statistics

For child self-report and parent proxy-report, all items were negatively skewed and 12 items showed skewness greater than -2. Table 1 presents the Cronbach's alphas, means, standard deviations, range, and percent of floor and ceiling effect of the PedsQL™ 4.0 Generic Core Scales for total sample. Cronbach's alpha coefficients for child self-report and parent proxy-report all exceeded the minimum reliability standard of .70. The alpha values were higher for the total score and lower for the school functioning scale of child self-report and parent proxy-report. Scale means all were higher than those of the PedsQL™ school study [3]. The full range of 0–100 was used for the emotional functioning scale of child self-report. The range of 40–100 was used for the total score and psychosocial health scale of parent proxy-report. There were essentially no floor effects. However, moderate to high ceiling effects existed in the majority of scales, except for the total score of child self-report. Especially, notable ceiling effects were found in the social functioning scale of child self-report and parent proxy-report in this mostly healthy sample.

**Table 1:** Scale descriptives for PedsQL™ 4.0 Generic Core Scales: Child self-report and parent proxy-report

Scale	Scale descriptives							
	
	Number of items	N	α	Mean	SD	Range	%Floor	%Ceiling
Child self-report								
Total Score	23	1396	.90	87.93	10.90	35.87–100	0.0	13.3
Physical Health	8	1405	.79	88.14	12.62	15.63–100	0.0	26.4
Psychosocial Health	15	1415	.87	87.73	11.72	20.00–100	0.0	18.6
Emotional Functioning	5	1418	.83	82.58	18.79	0.00–100	0.1	32.4
Social Functioning	5	1422	.82	93.47	11.31	25.00–100	0.0	60.8
School Functioning	5	1423	.72	87.07	13.10	20.00–100	0.0	30.6
								
Parent proxy-report								
Total Score	23	1399	.90	90.33	9.68	47.83–100	0.0	20.3
Physical Health	8	1415	.80	91.71	11.02	37.50–100	0.0	41.3
Psychosocial Health	15	1412	.88	89.52	10.64	43.33–100	0.0	25.4
Emotional Functioning	5	1422	.83	84.26	16.56	20.00–100	0.0	35.6
Social Functioning	5	1427	.88	89.31	12.40	15.00–100	0.0	69.3
School Functioning	5	1428	.75	89.29	12.39	30.00–100	0.0	41.3

α = Cronbach's alpha. % Floor/ceiling = percentage of scores at the extremes of the scaling range. Higher scores equal better health-related quality of life.

### Validity

Table 2 shows the goodness-of fit indices for four- and second-order factor model in the PedsQL™ 4.0 Generic Core Scales. All Chi-square statistics were significant and indicated a poor fit. For child self-report and parent proxy-report, the CFI approximated or exceeded the .90 standards of acceptable model fit and the TLI exceeded the .95 value of good model fit. For parent proxy-report ages 13–18, the CFI exceeded the .95 value of good model fit and the RMSEA was less than .08 that indicates a fair fit. For other scales, the RMSEA generally were greater than .08 but less than .09, those indicate a mediocre fit.

**Table 2:** Goodness-of-fit indices for four- and second-order factor model in the PedsQL™ 4.0 Generic Core Scales: Child self-report and parent proxy-report

Scale	Four-factor model					Second-order factor model				
		
	χ^2^	df	CFI	TLI	RMSEA	χ^2^	df	CFI	TLI	RMSEA
Child self-report										
Total (N = 1425)	1114.051*	98	.897	.961	.085	1055.771*	96	.902	.962	.084
Ages 8–12 (N = 633)	469.769*	92	.906	.955	.081	472.812*	92	.905	.955	.081
Ages 13–18 (N = 792)	535.513*	77	.934	.972	.087	490.199*	74	.940	.974	.084
										
Parent proxy-report										
Total (N = 1431)	826.681*	77	.942	.974	.082	806.168*	77	.944	.975	.081
Ages 8–12 (N = 638)	410.812*	68	.938	.968	.089	417.768*	68	.936	.967	.090
Ages 13–18 (N = 793)	399.522*	67	.959	.980	.079	376.245*	66	.962	.981	.077

CFI = Comparative fit index. TLI = Tuker-Lewis index. RMSEA = Root mean square error of approximation. *p < .00001.

Table 3 and 4 show the factor loadings and covariances for the four-factor and the second-order factor model across age group. As can be seen, all loadings are over .60, which indicates that the items and first-order factor fit well with their respective factors and their second-order factor. The covariances were relatively high, suggesting all scales are correlated across age group.

**Table 3:** Factor loadings of items for four-factor model in the PedsQL™ 4.0 Generic Core Scales: Child self-report and parent proxy-report

Factor and item	Child self-report			Parent proxy-report		
		
	Total	Ages 8–12	Ages 13–18	Total	Ages 8–12	Ages 13–18
Physical Health						
1. Hard to walk more than one block	.751	.749	.785	.809	.813	.808
2. Hard to run	.819	.784	.857	.867	.815	.902
3. Hard to do sports or exercise	.846	.785	.899	.872	.814	.913
4. Hard to lift something heavy	.746	.696	.805	.822	.791	.845
5. Hard to take a bath or shower	.623	.606	.683	.762	.762	.811
6. Hard to do chores around house	.694	.699	.718	.786	.795	.797
7. Hurt or ache	.719	.649	.769	.748	.757	.753
8. Low energy	.653	.642	.691	.670	.729	.678
Emotional Functioning	(.823)	(.818)	(.824)	(.840)	(.849)	(.835)
1. Feel afraid or scared	.794	.694	.872	.815	.800	.837
2. Feel sad or blue	.857	.786	.896	.872	.851	.889
3. Feel angry	.813	.751	.855	.813	.814	.815
4. Trouble sleeping	.724	.706	.738	.727	.690	.755
5. Worry about what will happen	.777	.714	.831	.796	.776	.821
Social Functioning	(.827)	(.856)	(.813)	(.833)	(.828)	(.841)
1. Trouble getting along with peers	.893	.833	.931	.910	.895	.925
2. Other kids not wanting to be friends	.889	.843	.928	.917	.915	.922
3. Teased	.699	.657	.766	.846	.833	.869
4. Doing things other peers do	.822	.823	.825	.896	.902	.900
5. Hard to keep up when play with others	.862	.836	.879	.894	.873	.907
School Functioning	(.722)	(.760)	(.726)	(.739)	(.732)	(.764)
1. Hard to pay attention	.806	.787	.813	.857	.836	.872
2. Forget things	.756	.682	.794	.784	.756	.807
3. Trouble keeping up with schoolwork	.780	.763	.784	.819	.822	.830
4. Miss school-not well	.838	.810	.858	.874	.886	.863
5. Miss school to go to doctor or hospital	.853	.832	.860	.892	.898	.879

Numbers in parentheses are factor loadings of subscale on psychosocial health of second-order factor.

**Table 4:** Covariances of factors for four factor model and second-order factor model in the PedsQL™ 4.0 Generic Core Scales: Child self-report and parent proxy-report

Scale and Factor	Total			Ages 8–12			Ages 13–18		
			
	Physical	Emotional	Social	Physical	Emotional	Social	Physical	Emotional	Social
Child self-report									
Psychosocial	(.799)			(.835)			(.786)		
Emotional	.672			.719			.656		
Social	.659	.666		.688	.690		.641	.658	
School	.559	.591	.621	.626	.576	.698	.555	.601	.607
									
Parent proxy-report									
Psychosocial	(.758)			(.733)			(.775)		
Emotional	.660			.668			.656		
Social	.622	.688		.581	.680		.651	.695	
School	.544	.608	.544	.507	.589	.658	.582	.637	.652

Numbers in parentheses are covariances between physical health factor and psychosocial health factor of second-order factor.

Table 5 contains the PedsQL™ 4.0 scores for healthy children and children with a chronic health condition within the sample. Consistent with previous findings [3,10] with the PedsQL™ 4.0, healthy children scored significantly higher on the PedsQL™ 4.0 (better HRQOL) than children with a chronic health condition in the scales. The only exception was on the social functioning scale of child self-report.

**Table 5:** Scale descriptives for PedsQL™ 4.0 Generic Core Scales child self-report and parent proxy-report: Healthy sample and chronic condition sample

Scale	Healthy sample			Chronic health condition sample			Difference	Effect size	t score
					
	N	Mean	SD	N	Mean	SD			
Child self-report									
Total Score	1341	88.16	10.74	48	81.23	13.53	6.93	0.65	-4.35***
Physical Health	1350	88.44	12.33	48	79.49	16.96	8.95	0.73	-4.87***
Psychosocial Health	1359	87.93	11.61	49	81.94	13.52	5.99	0.52	-3.53***
Emotional Functioning	1362	82.91	18.52	49	73.27	23.73	9.64	0.52	-3.55***
Social Functioning	1366	93.55	11.28	49	90.51	12.34	3.04	0.23	-1.85
School Functioning	1367	87.22	13.00	49	82.04	15.44	5.18	0.40	-2.72**
									
Parent proxy-report									
Total Score	1347	90.56	9.47	47	83.67	12.88	6.88	0.73	-4.83***
Physical Health	1362	92.02	10.75	48	82.62	14.87	9.40	0.87	-5.87***
Psychosocial Health	1360	89.70	10.49	47	84.08	13.49	5.62	0.54	-3.58***
Emotional Functioning	1370	84.43	16.40	47	78.40	20.14	6.03	0.37	-2.46*
Social Functioning	1373	94.97	10.15	49	91.63	12.05	3.34	0.33	-2.25*
School Functioning	1369	89.58	12.18	49	82.14	16.04	7.44	0.61	-4.15***

Effect sizes are designated as small (0.20), medium (0.50), and large (0.80). *p < .05. **p < .01.***p < .0001.

### Person and item reliability

Table 6 shows the reliability and separation index for persons and items across the four subscales. Person reliability and separation are low while Item reliability and separation are high. These results indicate that the sample has a narrow spread and the sample size is large enough.

**Table 6:** Reliability and separation index for PedsQL™ 4.0 Generic Core Scales: Child self-report and parent proxy-report (Total sample only)

Scale and index	Child self-report		Parent proxy-report	
		
	Person	Item	Person	Item
Physical Health				
Reliability	.54	1.00	.49	.99
Separation	1.09	15.71	.99	13.81
Emotional Functioning				
Reliability	.59	.99	.60	.99
Separation	1.20	9.58	1.24	12.67
Social Functioning				
Reliability	.40	.95	.59	.97
Separation	.82	4.44	1.20	5.56
School Functioning				
Reliability	.45	1.00	.44	1.00
Separation	.91	19.55	.89	16.78

### Item statistics

Table 7 shows item infit and outfit statistics on the four subscales. The majority of items showed mean square infit and outfit statistics within the 0.6 and 1.4 range, save for item 5 (Hard to take a bath or shower) of the physical health scale and item 3 (Teased) of the social functioning scale for child self-report.

**Table 7:** Item statistics for PedsQL™ 4.0 Generic Core Scales: Child self-report and parent proxy-report (Total sample only)

Factor and item	Child self-report		Parent proxy-report	
		
	Infit MNSQ	Outfit MNSQ	Infit MNSQ	Outfit MNSQ
Physical Health				
1. Hard to walk more than one block	1.00	.73	.97	.59
2. Hard to run	.89	.87	.95	.84
3. Hard to do sports or exercise	.88	.71	.96	.88
4. Hard to lift something heavy	.92	.92	.81	.75
5. Hard to take a bath or shower	1.43	1.00	1.15	1.06
6. Hard to do chores around house	1.08	.99	1.01	1.00
7. Hurt or ache	1.16	1.05	1.19	.99
8. Low energy	1.29	1.26	1.33	1.30
Emotional Functioning				
1. Feel afraid or scared	.92	.92	.99	.98
2. Feel sad or blue	.79	.75	.73	.70
3. Feel angry	.93	.93	.95	.93
4. Trouble sleeping	1.34	1.28	1.32	1.29
5. Worry about what will happen	1.09	1.10	1.07	1.11
Social Functioning				
1. Trouble getting along with peers	.77	.77	1.00	.99
2. Other kids not wanting to be friends	.76	.80	.81	.80
3. Teased	1.44	1.41	1.22	1.23
4. Doing things other peers do	1.08	1.11	.97	1.05
5. Hard to keep up when play with others	.93	.92	1.02	.95
School Functioning				
1. Hard to pay attention	.84	.84	.86	.85
2. Forget things	1.03	1.01	1.07	1.06
3. Trouble keeping up with schoolwork	1.08	.97	.95	.93
4. Miss school-not well	1.17	1.17	1.15	1.07
5. Miss school to go to doctor or hospital	1.10	.97	1.14	1.08

Infit = Information-weighted fit statistic. Outfit = Outlier-sensitive fit statistic. MNSQ = Mean-square statistic with expectation 1.

### Rating scale diagnostics

Table 8 shows average measures, infit and outfit MNSQ, and step measures on the four subscales. The average measures in all scales of child self-report and parent proxy-report increase monotonically across the rating scale. They function as expected and indicate that, on average, persons with higher measures selected higher categories. Most infit and outfit are close to 1.00 or a little below except category 4. The people who chose each category accord with the people we would expect to choose those categories. Somewhat problematic is the infits or the outfits for category 4 in the physical, social and school functioning of child self-report and all subscales of parent proxy-report. This indicates that persons with low measures unexpectedly selected this high category. Step measures indicate the structure of the category probability curves in as sample-independent manner as possible. They are advancing, and show a structure of a "range of hills" in physical, emotional, and school functioning of child self-report and parent-proxy-report. However, step measures 3 and 4 are disordered in social functioning of child self-report and parent proxy-report.

**Table 8:** Category measures and fit for PedsQL™ 4.0 Generic Core Scales: Child self-report and parent proxy-report (Total sample only)

Scale and category label	Child self-report				Parent proxy-report			
		
	Average Measure	Infit MNSQ	Outfit MNSQ	Step measure	Average measure	Infit MNSQ	Outfit MNSQ	Step measure
Physical Health								
0	-29.26	1.01	1.01	None	-32.57	1.02	1.02	None
1	-16.10	.97	.69	-12.66	-18.64	.90	.63	-14.97
2	-9.31	1.04	1.02	-8.91	-10.51	1.04	.97	-11.26
3	-1.81	1.10	1.21	7.61	-3.90	1.33	1.60	9.84
4	1.99	1.53	1.59	13.97	-.10	1.76	2.13	16.29
Emotional Functioning								
0	-23.63	1.02	1.02	None	-32.14	.98	.99	None
1	-14.99	.92	.90	-15.95	-20.52	.98	.99	-24.84
2	-7.05	.97	.96	-8.62	-9.41	.95	.96	-12.54
3	1.52	.92	.98	8.25	1.94	1.04	1.07	13.31
4	6.37	1.33	1.34	16.32	2.91	1.93	2.01	24.07
Social Functioning								
0	-29.17	1.02	.99	None	-43.57	.98	.92	None
1	-19.01	.90	.93	-21.40	-25.79	.86	.91	-35.72
2	-9.12	1.02	1.03	-1.46	-9.49	1.10	1.14	-.13
3	-1.28	1.03	1.06	12.34	7.99	.93	1.03	21.80
4	2.73	1.30	1.54	10.52	9.87	1.94	3.23	14.05
School Functioning								
0	-36.90	1.03	1.02	None	-39.70	.99	.99	None
1	-20.95	.94	.91	-23.84	-23.79	.91	.85	-27.76
2	-9.14	1.01	1.03	-8.79	-11.59	1.05	1.15	-10.98
3	1.84	.97	.98	13.00	-1.87	1.14	1.16	13.86
4	8.93	1.38	1.43	19.63	3.83	1.40	1.45	24.89

Infit = Information-weighted fit statistic. Outfit = Outlier-sensitive fit statistic. MNSQ = Mean-square statistic with expectation 1. 0 = never a problem. 1 = almost never a problem. 2 = sometimes a problem. 3 = often a problem. 4 = almost always a problem.

**Figure 1:**
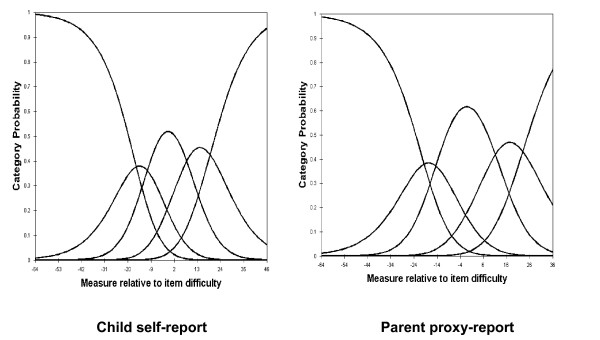
Response Functions for 5 categories: Physical Health.

**Figure 2:**
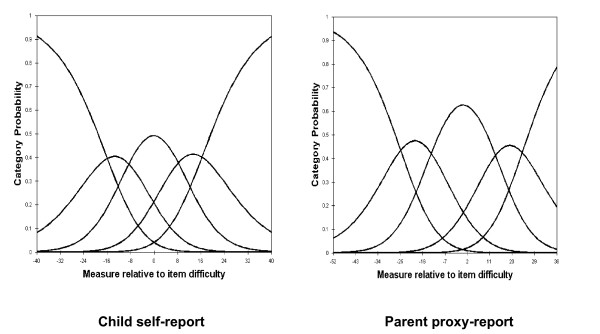
Response Functions for 5 categories: Emotional Functioning.

**Figure 3:**
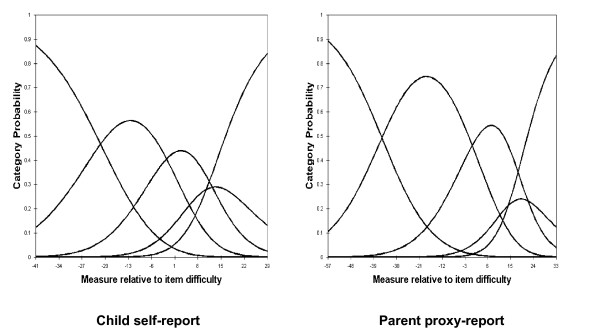
Response Functions for 5 categories: Social Functioning.

**Figure 4:**
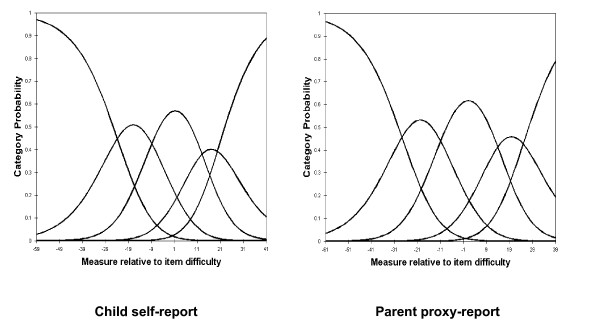
Response Functions for 5 categories: School Functioning.

For child self-report and parent proxy-report, the RSM category probability curves are shown in Figures 1, 2, 3 and 4. There are 5 curves visible for each scale, starting from the left. They in general depict the expected succession of "hills". However, the disordered step measures 3 and 4 in social functioning scales of child self-report and parent proxy-report also are reflected in the probability curves. As shown in Figure 3, the cross-over between the curves for category 3 and 4 is to the left of that for category 2 and 3 in social functioning scales of child-self-report and parent proxy-report.

### Parent/child agreement

Table 9 shows the ICCs between PedsQL™ 4.0 child self-report and parent proxy-report. For the total sample, ICCs were higher for total score and psychosocial health scales and lower for physical health scale. For children ages 8–12, ICCs were higher for school functioning scale and lower for physical health scale and social functioning scale. For adolescents ages 13–18, ICCs were higher for total score and psychosocial health scale and lower for physical health scale and social functioning scale. However, the range of ICCs was between .47 and .61 across the ages. These results suggest moderate agreement. In particular, there was good agreement for the total score of ages 13–18. Furthermore, the results indicate a trend towards higher ICCs with increasing age, save for the school functioning scale.

**Table 9:** Agreement between PedsQL™ 4.0 Generic Core Scales for parent proxy-report and child self-report across scales and ages 8–18

Scale	Age Group		
	
	Total Sample	Child (8–12)	Adolescent(13–18)
Total Score	.58	.54	.61
Physical Health	.49	.47	.50
Psychosocial Health	.58	.54	.60
Emotional Functioning	.55	.50	.57
Social Functioning	.50	.48	.50
School Functioning	.56	.59	.54

Values are Single Measure Intraclass Correlation Coefficients (ICC). ICC values were derived using a two way mixed effects model with absolute agreement type. ICC are designated as ≤ 0.40 poor to fair agreement, 0.41–0.60 moderate agreement, 0.61–0.80 good agreement, and 0.81–1.00 excellent agreement.

## Discussion

The purpose of this study was to assess the psychometric properties of the Korean translation of the PedsQL™ 4.0 Generic Core Scales in school children and adolescents ages 8–18. Like in the school study with the original U.S. English instrument [3] and other translation studies [4,42,43], items on the PedsQL™ 4.0 had minimal missing responses. It suggests that children and parents are willing and able to provide good quality data regarding the child's HRQOL [3].

There were no floor effects and moderate to high ceiling effects, especially for social functioning scales, which showed notable ceiling effects. These findings might be expected for a healthy school-age population. Responsiveness is an important measurement property in a clinical trial, and one of the factors that can affect responsiveness is floor and ceiling effect [19]. However, detecting improving health among persons who are already quite well may prove difficult because of ceiling effects, and most school children are quite healthy [3]. The presence of ceiling effects may be expected in generic HRQOL instruments since they are designed to be applicable to a wide range of populations [44]. Thus, the findings can be a reflection of the sample characteristics, i.e., a healthy school population. Although most children are quite healthy, measuring HRQOL in large school populations has several distinct benefits. It can aid in identifying subgroups of children who are at risk for health problem, in determining the burden of a particular disease or disability, and informing efforts aimed at prevention and intervention [45]. In addition, utilization of HRQOL measures may assist in the evaluation of the healthcare needs of a school district, and results can be used to inform public policy, including the development of strategic healthcare plans and school health clinics, identifying health disparities, promoting policies and legislation related to school health, and aiding in the allocation of health care resources [46].

On the other hand, it has been suggested that concepts and measures from the more positive end of the HRQOL continuum are needed for healthy populations [47] and inclusion of emotional well-being, positive affect, vitality, and health perceptions aid in discriminating and measuring change in well populations [48]. Even though the items of PedsQL™ 4.0 are reverse-scored and higher score indicate better HRQOL, the instructions ask how much of a problem each item has been during the past 1 month. In other words, the interaction between sample characteristics and the focus on "problems" in the items and instructions of PedsQL™ 4.0 might cause such ceiling effects in a healthy sample. Finally, in the Korean culture, individuals who have good interpersonal relationships tend to be regarded as having a good personality and virtue, which may lead to some social desirability responding on social functioning items, leading to notable ceiling effects. Compared with other translation studies [43,49], these potential cultural differences require further research using a wide range of the Korean population, including healthy and chronically ill children and adolescents to more fully understand cultural differences.

The CFA on the PedsQL™ 4.0 Generic Core Scales supported a four-factor model and a second-order factor model. It suggests the statistical evidence that the PedsQL™ 4.0 Generic Core Scales cover the core dimensions of health as delineated by the WHO and have construct validity for the utilization of five summary and scale scores.

Children with chronic health conditions were reported to experience lower physical, emotional, and school functioning in comparison to healthy children. This indicates that PedsQL™ 4.0 Generic Core Scales can differentiate HRQOL in healthy children as a group in comparison to children with chronic health conditions. However, there was no significant difference on the social functioning scale between healthy and unhealthy children in this study, even though the social functioning of the children with chronic health conditions was lower than that of the healthy children. In the previous PedsQL™ school study in the US [3], there was a statistically significant difference on the social functioning scale between healthy and unhealthy children. Comparisons to the mean scores of the other subscales within the present study to those of the previous PedsQL™ school study [3], the mean scores on the social functioning scale of both healthy children and unhealthy children were very high. Therefore, non-significant difference on the social functioning of child self-report should be further studied in Korean samples, especially when compared to clinical populations with larger sample sizes of chronically ill children with physician-diagnosed chronic health conditions. This comparison is essential because the type and severity of chronic health conditions did not have a significant impact on the social functioning of the children who participated in the present study. In addition, it should be noted that it might be caused by social desirability and cultural differences in Korean populations.

Rasch RSM analysis on the four subscales of PedsQL™ 4.0 Generic Core Scales show that person reliability and separation are low, while item reliability and separation are high. As we mentioned earlier, these results indicate that the sample has a narrow spread and the sample size is large enough. Person reliability refers to the replicability of person placement across other items measuring the same construct. Item reliability refers to the replicability of item placement within the hierarchy across other samples [28]. The chief influences on person reliability are sample "true" standard deviation, test length, number of categories per item, and test targeting sample [50]. In this study, test lengths of each subscale are adequate in length and number of categories per item is sufficient. Person reliability is a characteristic of the person measures for the sample being tested. To increase person reliability, testing persons with more extreme abilities or attitudes and improving the test targeting may be slightly helpful. PedsQL™ 4.0 Generic Core Scales have been originally developed for targeting clinical samples. Considering the predominantly healthy characteristics of this study sample, most of the PedsQL™ 4.0 Generic Core Scales items might be too "severe" for healthy school populations in Korea. On the other hand, it should be noted that internal consistency reliability alpha coefficients presented in Table 1 were between .72 and .90. However, raw-score based reliabilities (e.g., Cronbach's alpha) in general overstate the "true" reliability while the Rasch reliabilities understate the "true" reliability [51]. Therefore, further studies on clinical samples are needed to find out what exactly caused low person reliability in Korean samples. According to the results of item statistics, all items of the subscales were found to represent a homogenous construct and it has been already confirmed by CFA as well. Rating scale diagnostics to identify the optimal categorization showed that category 4 is somewhat problematic as well as step measures 3 and 4 are disordered in the social functioning scale of child self-report and parent proxy-report. These results indicate a low probability of observance of certain categories, i.e., category 4 (almost always a problem) seems not to work as intended for this healthy school sample in Korea.

The pattern of parent-child correlation for the total sample, child ages 8–12, and adolescent ages 13–18 was different from those of the PedsQL™ 4.0 school population study [3] and the UK-English version study on the PedsQL™ 4.0 Generic Core Scales [49], where better correlation was found for physical than for psychological and social functioning. While it might be expected that the intercorrelations between child and parent report across the physical, emotional, social and school functioning scales would follow the conceptualization that more observable domains (i.e., physical functioning) would yield higher agreement, this has not necessarily been the case in the published literature with other HRQOL instruments. A comprehensive review [52] found mixed results in terms of higher intercorrelations between self and proxy reports of physical functioning across pediatric HRQOL instruments, with most studies demonstrating this effect, while some others did not. In addition, it was suggested that levels of agreement can be affected by child age, domains investigated, and parent's own QOL [40]. On the other hand, all the ICCs between PedsQL™ 4.0 child self-report and parent proxy-report showed moderate agreement and a general trend towards higher agreements with increasing age. The ICCs were consistently higher than those of the PedsQL™ 4.0 school population study [3], despite the fact that the ICC values of this study were derived using absolute agreement type while the PedsQL™ 4.0 school population study used consistency type. In situations where children are unable or unwilling to respond for themselves, measurement of QOL is often obtained by parent proxy-report [40]. Thus, these consistencies between child self-report and parent proxy-report suggest that parent proxy-report can be informative for measuring HRQOL of children when they are not able to respond. The trend towards higher agreement with increasing age is consistent with the results of the PedsQL™ 4.0 school population study [3] and can be explained by the greater verbal communication skills typically manifested with increasing developmental age.

There are several limitations to this study. First, we were not able to collect data from a representative sample based on the Korean population census. However, it should be noted that we had a large enough sample size in two small cities, two metropolitan cities, and a capital city. Second, we were not able to determine which children and adolescents did not understand the instructions of PedsQL™ 4.0 due to cognitive dysfunction, even though there were no developmental disorders in the parent's report on the presence of a chronic health condition in their children. For this study, PedsQL™ 4.0 Generic Core Scales were administered as a group test in schools, and thus, there may be some covariates that were not accounted for. Furthermore, the sample size for children with a chronic health condition was very small, and may not be representative of chronically ill children in general or specifically in Korea. In particular, if the same factor structure is not confirmed on a less healthy population, their scores might not be comparable. Thus, further validation studies on Korean clinical samples are required. Finally, we applied the unidimensional Rasch model to analyze item responses in the PedsQL™ 4.0 Generic Core Scales. However, the unidimensional approach ignores the correlations between latent traits and yields imprecise measures when tests are short [53]. PedsQL™ 4.0 Generic Core Scales can be analyzed as a whole, but the approach ignores the evidence for the subscale structure. In a further study, to take the correlations into account, the application of multidimensional item response models is needed. Additionally, for assessing cross-cultural equivalence of PedsQL™ 4.0 Generic Core Scales, the analysis of differential item functioning (DIF) is needed for both the Korean and the US samples.

## Conclusion

The results demonstrate the feasibility, validity, item reliability, item fit, and agreement between child self-report and parent proxy-report of the Korean version of PedsQL™ 4.0 Generic Core Scales for school population health research in Korea. However, the utilization of the Korean version of the PedsQL™ 4.0 Generic Core Scales for healthy school populations needs to consider low person reliability, ceiling effect and cultural differences, and further validation studies on Korean clinical samples are required.

## Abbreviations

HRQOL: Health-Related Quality of Life; PedsQL™: Pediatric Quality of Life Inventory™; WHO: World Health Organization; CTT: Classical Test Theory; IRT: Item Response Theory; CFA: Confirmatory Factor Analysis; WLSMV: Weighted Least Square Parameter Estimates Using a Diagonal Weighted Matrix with Standard Errors and Mean-and Variance-Adjusted Chi-Square Test Statistic; CFI: Comparative Fit Index; TLI: Tuker-Lewis Index; RMSEA: Root Mean Square Error of Approximation; PCM: Partial Credit Model; RSM: Rating Scale Model; ICC: Intraclass Correlation; INFIT: Information-Weighted Fit Statistic; OUTFIT: Outlier-Sensitive Fit Statistic; MNSQ: Mean-Square Statistic with Expectation 1; DIF: Differential Item Functioning.

## Competing interests

Dr. Varni holds the copyright and the trademark for the PedsQL™ and receives financial compensation from the Mapi Research Trust, which is a nonprofit research institute that charges distribution fees to for-profit companies that use the Pediatric Quality of Life Inventory™. The PedsQL™ is available at the PedsQL™ Website [18].

## Authors' contributions

SHK and JWV designed the study, SHK collected the data and performed the statistical analyses, SHK and JWV drafted the manuscript, JWV participated in the statistical analyses. All authors read and approved the final manuscript.
